# GASP/WFIKKN Proteins: Evolutionary Aspects of Their Functions

**DOI:** 10.1371/journal.pone.0043710

**Published:** 2012-08-24

**Authors:** Olivier Monestier, Caroline Brun, Olivier Cocquempot, Daniel Petit, Véronique Blanquet

**Affiliations:** 1 INRA, UMR1061 Unité de Génétique Moléculaire Animale, Limoges, France; 2 Université de Limoges, Limoges, France; Ecole Normale Supérieure de Lyon, France

## Abstract

Growth and differentiation factor Associated Serum Protein (GASP) 1 and 2 are proteins known to be involved in the control of myostatin activity at least *in vitro*. Most deuterostome GASPs share a modular organization including WAP, follistatin/kazal, IGc2, two kunitz, and NTR domains. Based on an exon shuffling model, we performed independent phylogenetic analyses on these modules and assessed that papilin is probably a sister sequence to GASP with a divergence date estimated from the last common ancestor to bilateria. The final organization was acquired by the addition of the FS domain in early deuterostomes. Our study revealed that *Gasp* genes diverged during the first round of genome duplication in early vertebrates. By evaluating the substitution rate at different sites on the proteins, we showed a better conservation of the follistatin/kazal domain of GASP1 than GASP2 in mammals, suggesting a stronger interaction with myostatin. We also observed a progressive increase in the conservation of follistatin and kunitz domains from the ancestor of *Ciona* to early vertebrates. *In situ* hybridization performed on mouse embryos showed a weak Gasp1 expression in the formed somites at 10.5 dpc and in limb buds from embryonic E10.0 to E12.5. Similar results were obtained for zebrafish embryos. We propose a synthetic view showing possible interactions between GASP1 and myostatin and highlighting the role of the second kunitz domain in preventing myostatin proteolysis.

## Introduction

GASP1 ( =  WFIKKN2) and GASP2 ( =  WFIKKN1), are two highly similar multi-domain secreted proteins including several modules retrieved in protease-inhibitory proteins: a signal peptide, a whey acidic protein domain (WAP), a follistatin/kazal domain, an immunoglobulin domain, two tandem kunitz modules and a NTR domain [1], [2]. Currently, only the second kunitz domain has been proved to have a functional antiprotease activity [3]. *In vitro* experiments [4] showed that these two proteins have a high affinity for two TGF beta family members encoded by paralogous genes and implicated in musculoskeletal development: myostatin also known as GDF8 (growth and differentiation factor 8), a protein playing an inhibitory role in prenatal and postnatal muscle development, and bone morphogenic protein 11 also called GDF11 which is implicated in axial skeleton formation during embryogenesis [5], [6]. GDF8 and GDF11 are synthesized as precursor proteins composed of a signal sequence, an N-terminal propeptide domain and a C-terminal active domain. After cleavage by a furin protease, the propeptide remains non-covalently attached to the active proteins in an inactive latent complex. The active protein is released by further proteolytic cleavages and degradation of the propeptide [7]. The binding affinity between the GASP and GDF8/GDF11 proteins is essentially due to the follistatin/kazal domain [4]. GASP proteins can also bind to the myostatin propeptide, implicated in GDF8 inhibition by protein-protein interaction, due to their NTR domain hypothetical fixation [2], [4]. The binding of GASP to GDF8 has an *in* *vitro* effect on its affinity for the membrane receptor ActRIIB [2]. Morevover, Haidet *et* *al*. [8] observed a significant increase in skeletal muscle mass and strength in mice after cytomegalovirus-*Gasp1*-AAV1 (Adeno-associated virus 1) muscle injection. Taken together, these results suggest the implication of GASP proteins in the regulation of muscle mass in a myostatin dependent manner. Interestingly, if *Gdf8* and *Gdf11* expression is restricted to specific organs (muscle, axial skeleton), *Gasp1* and *Gasp2* are expressed in numerous tissues including during fetal development: brain, skeletal muscle, thymus and kidney for *Gasp1*, and lung, skeletal muscle and liver for *Gasp2*. In the adult human, *Gasp1* is expressed in ovary, testis, pancreas, brain, lung, and *Gasp2* in pancreas, thymus, liver, kidney, lung, testis and inner ear [1], [4], [9]. These expression patterns argue for physiological roles independent of interactions with GDF8 and GDF11. Supporting that view, it has been recently shown that both GASP proteins can bind to TGF beta1, BMP2 and BMP4 but do not inhibit their activity *in* *vitro* [10].

Little is known about GASP evolution, except a sequence in urochordate (*Ciona intestinalis*) orthologue to the two proteins present in all the vertebrates [4], [11]. To better understand the role of GASP, we studied the evolutionary changes in their function to determine (i) how the GASP modular organization emerged during metazoan evolution, (ii) if the divergence between both *Gasp* genes was linked to the two rounds of genome duplication in early vertebrates, and (iii) the conservation and variation at the different domains.

## Results

### Evolutionary Origin of GASP1 and GASP2

**Figure 1:**
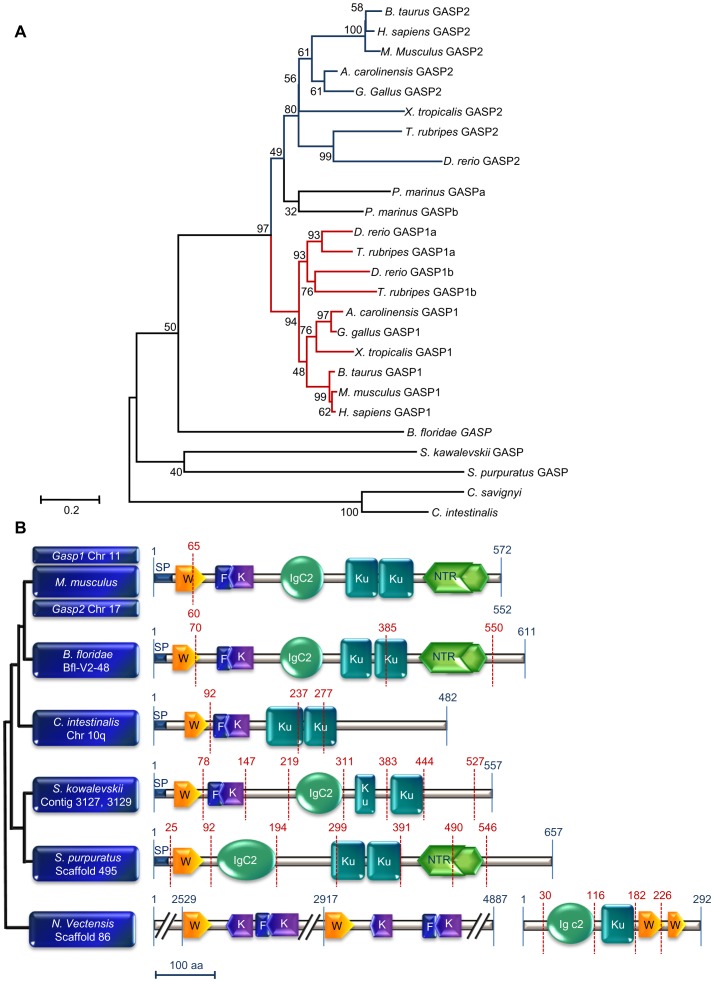
Evolution of GASP proteins. A) Phylogeny of deuterostome sequences obtained from maximum likelihood method using JTT as transition matrix with the option +G, +I and 500 replicates. Sequences related to GASP1 and GASP2 are in red and blue respectively. Bootstrap values are indicated above the branches. The access number of each sequence is indicated in Tables S1, S2 and S3. B) Comparison of the different domains of GASP in deuterostomes. The vertical red lines indicate the position of introns in the encoding genes. Abbreviations: SP: signal peptide, W: WAP, F: follistatin, K: kazal, Ku: kunitz, NTR: netrin domains.

GASP1 and GASP2 are proteins retrieved in every group of vertebrates, but there are two members of GASP1 (GASP1a, GASP1b) in *Danio rerio* and in a few other fish species. Protein length varies between 482 (*Ciona intestinalis*) and 657 amino acids (sea urchin *Strongylocentrotus purpuratus)*. The mouse sequences are 571 and 552 amino acids long for GASP1 and GASP2 respectively. The phylogeny of deuterostome proteins orthologous to GASP was first deduced from maximum likelihood methods. Using Gblocks selected sites, ProtTest program [12] revealed that the best fit model was JTT+G (Gamma distributed in 4 classes) +I (invariant sites). As seen in Figure 1A, the phylogenetic tree showed the homogeneity on one hand, between vertebrate GASP1 sequences supported by a high bootstrap value and on the other hand, between vertebrate GASP2 sequences but with a value close to 50%. In the lamprey *Petromyzon marinus*, although three sequences were retrieved and found to be related to GASP2, only the two complete ones were considered in the phylogenetic tree. We tested other alternatives to assess the position of *P. marinus* sequences, by considering other options with maximum likelihood or minimum evolution methods (Table 1). The same topology as previously with bootstrap values around 50% was found. Using the entire alignment, we obtained similar results with a better bootstrap value (Table 1). Therefore, our data suggest that both lamprey sequences are related to GASP2 and would not be phylogenetically basal to both GASP subfamilies. Within deuterostomes, there was only one sequence related to the ancestor to GASP1 and GASP2. The *Branchiostoma floridae* GASP sequence was closer to vertebrate counterparts than to the *Ciona* sequences, a topology supporting the phylogeny hypothesized by Adoutte *et al.* [13] and Cameron *et al.* [14] but challenged by Philippe *et al.* [15]. It should be noted that the common deletion approximately 80 aa inside the IGc2 domain in both *Ciona* species (*C*. *intestinalis* and *C. savignii*) could play a role in the peripheral position of *Ciona* sequences.

**Table 1:** Bootstrap values supporting that lamprey sequences belong to the GASP2 branch.

	method	model	rate among site	bootstrap value
Gblocks selected sites (407 sites)	ML	JTT	+G+I	49
		JTT	+G	46
		WAG	+G+I	46
	ME	JTT	+G	51
complete alignment (999 sites)	ML	JTT	+G	71
		JTT	+G+I	71
	ME	JTT	+G	67

ML: Maximum likelihood, ME: Minimum Evolution.

The modular organization of GASP proteins already described in mammals was similar in *Branchiostoma floridae* (Fig. 1B). In both *Ciona*, the IGc2 domain is lacking and the NTR domain is not recognized in the C-terminal part of the protein. Interestingly, except for the follistatin domain in sea urchin and the netrin domain in acorn worm, all modules are present in this clade. Taken together, this data indicates that GASP modular organization present in vertebrates was already acquired in the common ancestor to sea urchin, acorn worm and chordates.

In spite of a thorough search of protostome genomes, no trace of GASP was retrieved. Instead, scaffold 86 of the sea anemone *Nematostella vectensis* contains two genes encoding proteins harboring the WAP and follistatin modules (4887 aa) as well as the IGc2, kunitz and WAP domains (292 aa). To decipher the events that occurred between these probable ancestors and the present deuterostome organization, we performed (i) an exhaustive search of the mouse genome for proteins including at least one domain in common with GASP, and (ii) a survey of proteins sharing at least two different modules found in GASP. Then, separate phylogenies of the main modules (WAP, IGc2, kunitz and kazal) were built. WAP phylogeny indicated that the papilin found in *D. melanogaster* protostomes and in the worm *Capitella teleta* contained a WAP closely related to GASP (Fig. S1). The absence of this domain in deuterostome papilins may correspond to a loss. Some proteins with only one or two WAPs (EXPI, WFDC2 for example) are situated between the papilin/GASP group and the two proteins present in *Nematostella vectensis*. Another group containing a WAP and a kunitz (Eppin and WAP8C for example) was found to be distantly related to the previous groups. For the kazal domains, the closest to GASP are those from the tomoregulin isoform 2, agrin and follistatin-like proteins (FSTL1, FSTL4 and FSTL5) (Fig. S2). The 18 kazal domains included in the 4887 aa protein present in *Nematostella vectensis* were close to the kazal domains included in follistatin and in follistatin-like 3. Kunitz phylogeny pointed out that both GASP kunitz domains were very distantly related (Fig. S3). The N-terminal kunitz of this protein was close to the 4^th^ and 9^th^ kunitz of *Drosophila melanogaster* papilin, the 3^rd^ one of sea urchin XP_789144, proteins constituted of 1 to 3 kunitz (kunitz 5, SPIT4, TFPL), and WAP8C. The C-terminal kunitz of GASP was related to the 2^nd^ and 4^th^ kunitz of sea urchin and 3^rd^ kunitz of *D. melanogaster* papilin, to Eppin, WFDC6B, Q3UW55 and to Spint3-like. The kunitz present in the 292 aa protein of *Nematostella* was situated at the same distance from the two previous groups, which is compatible with its ancestral significance. IGc2 phylogeny, mostly studied for proteins containing at least two modules present in GASP, argues that GASP, papilin and follistatine-L are related proteins. Interestingly, IGc2 present in the 292 aa protein of *Nematostella* was close to GASP counterpart (Fig. S4). Although ADAMTS-L1 shared only IGc2 domain with previous proteins, the 3^rd^ was close to the 3^rd^ IGc2 of *D. melanogaster* papilin.

In most vertebrate species, the genes corresponding to GASP1 and GASP2 are organized in two exons and one intron inside the portion encoding the WAP domain. In *Branchiostoma* and *Ciona*, there are two extra introns, one shared by both species in the region encoding the second kunitz domain, and one proper to a species near the 3′ coding sequence end in *Branchiostoma* and within the part encoding the first kunitz in *Ciona*. In acorn worm and sea urchin, the nucleotide sequences corresponding to most domains are separated by introns. In *Nematostella*, the different domains in the smaller protein are also encoded by separated exons.

### Paralogy and Synteny Analyses

**Figure 2:**
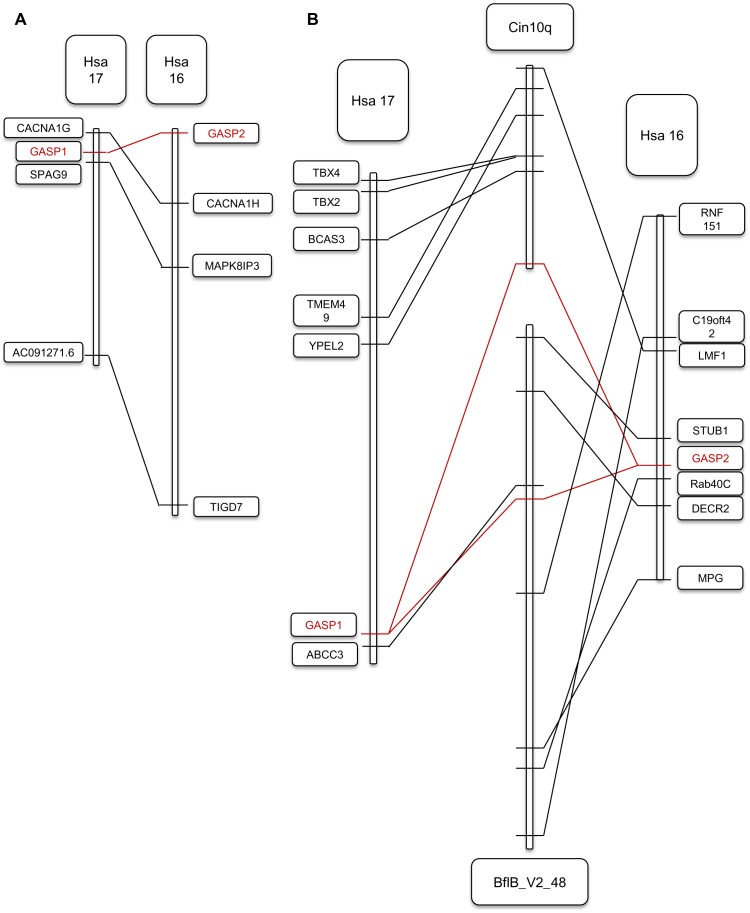
Synteny and paralogy of *Gasp*. A) Paralogons situated on human chromosomes 16 and 17 (HSA16; HSA17) including both *Gasp* genes. B) Orthology between each paralogon and chromosome 10q of *Ciona* and the contig 48 of *Branchiostoma*.

To test if the duplication of *Gasp1* and *Gasp2* was true for their respective neighbouring genes, we searched for paralogons (blocks of paralogs) in the human genome using a paralogon website [16] and synteny database [17]. Three paralogous genes shared the same duplication history as *Gasp* when the window size was 50, *Branchiostoma floridae* taken as an outgroup: *Cacna1G/H*, *Mapk8IP3/Spag9* and *TIGD7/Ac091271.8* (Fig. 2A). If we extended the size of the window to 100, we found 42 genes. In addition, two series of genes surrounding human *Gasp1* and *Gasp2* had orthologues in *Ciona intestinalis* chromosome 10q and in the scaffold BflB_V2_48 of *B. floridae* (Fig. 2B).

### Variation in Substitution Rates

**Figure 3:**
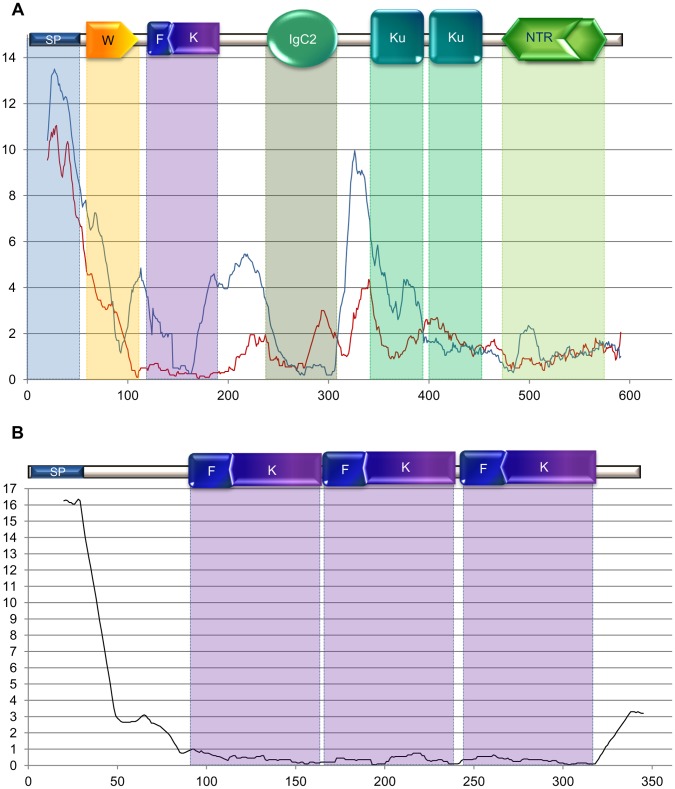
Substitution rate within GASP proteins. For each site the substitution rate was calculated from a moving average using a window size of 20 amino acids. The site position from start-methionine is on the abscissa and the mean substitution rate on the ordinate. A) Substitution rate in mammalian GASP. The red and blue curves correspond to GASP1 (23 species) and GASP2 (25 species) respectively. B) Substitution rate in mammalian follistatin (22 species). Abbreviations: SP: signal peptide, W: WAP, F: follistatin, K: kazal, Ku: kunitz, NTR: netrin domains.

The analysis of substitution rates along the proteins in more than 20 mammalian species (Fig. 3A) has indicated parallel variations at a higher level in GASP2 than in GASP1. The main peaks were observed in the N-terminal signal peptide and at the junctions of kazal/IGc2 and IGc2/kunitz domains. The most conserved domains were found in the netrin of both proteins, the follistatin/kazal module for GASP1 and the IGc2 module for GASP2. Otherwise, there was a higher degree of conservation in the second kunitz than in the first one for GASP2. A similar study was undertaken for the three follistatin/kazal domains of mammalian follistatin (Fig. 3B). The substitution rate level was very low and quite comparable to that observed in the same domain in GASP1.

**Figure 4:**
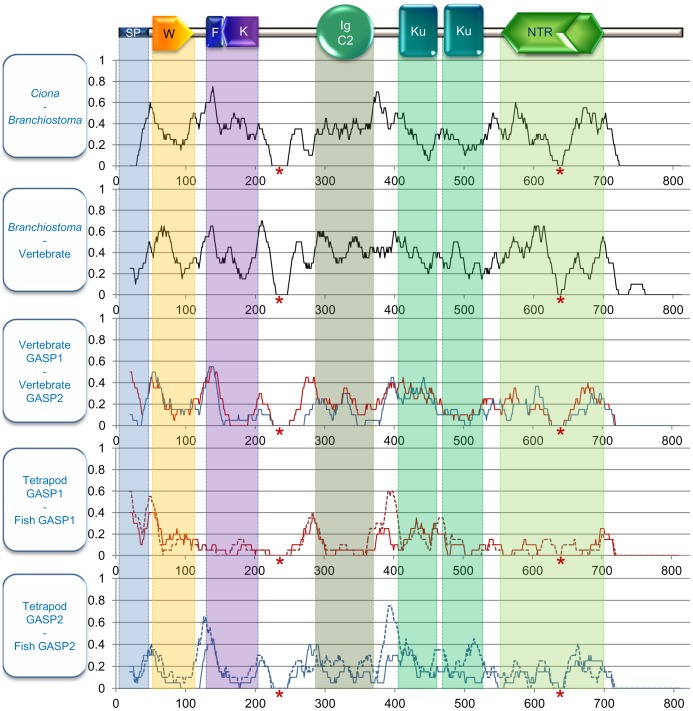
Substitution rate of GASP proteins in the different branches of the evolutionary tree. For each site the substitution rate was calculated from a moving average using a window size of 20 amino acids. The site position from start methionine is on the abscissa and the mean substitution rate on the ordinate. The black curves correspond to the ancestor of GASP1 and GASP2 proteins. The red and blue curves correspond to GASP1 and GASP2 respectively. Red asterisks represent gaps in sequence alignment due to one or two species. In the last two parts, the dotted lines correspond to fish and the solid lines to tetrapods. Abbreviations: SP: signal peptide, W: WAP, F: follistatin, K: kazal, Ku: kunitz, NTR: netrin domains.

We then compared the variations of substitution rate in the different branches from *Ciona* to tetrapods and fish GASP1 and GASP2 (Fig. 4). In the WAP domain, the C-terminal half was always more substituted than the second half. In the follistin/kazal domain, the previous observation of high conservation for mammal GASP1 was again seen in the branches leading to fish and tetrapods GASP1, while the profiles in other cases were similar to that observed in mammalian GASP2. In the IGc2 domain, there was a slightly decreased substitution rate from the *Ciona* ancestor to tetrapods and fish GASP1 and GASP2 branches. The C-terminal half of the first kunitz domain was more variable than the second half especially in GASP1 and GASP2 fish sequences. In contrast to what was observed in mammalian GASP2, the second kunitz sequences did not seem to be more conserved than the first one during the evolution of Deuterostomes. In the netrin domain the sequence variability decreased from the *Ciona* ancestor to vertebrate GASP1 and GASP2.

### Expression of *Gasp1* in Mouse and Zebrafish Embryos

Expression domains were visualized by *in situ* hybridization of endogenous transcripts of *Gasp1* on 9.5 dpc to 12.5 dpc mouse embryos. At 9.5 dpc, *Gasp1* was expressed in the neural tube. At 10.5 dpc, expression extended to the limb buds and somites. At 11.5 dpc, while *Gasp1* expression in somites became very low, it could be detected in the caudal part and the proximo-distal axis of embryo. At 12.5 dpc, staining was restricted to the limbs, genital bud and caudal region. Within the limbs, *Gasp1* was expressed in the anterior and posterior part of the bud at 11.5 dpc. Later at 12.5 dpc, this expression extended to precartilaginous condensations that give rise to fingers.

**Figure 5:**
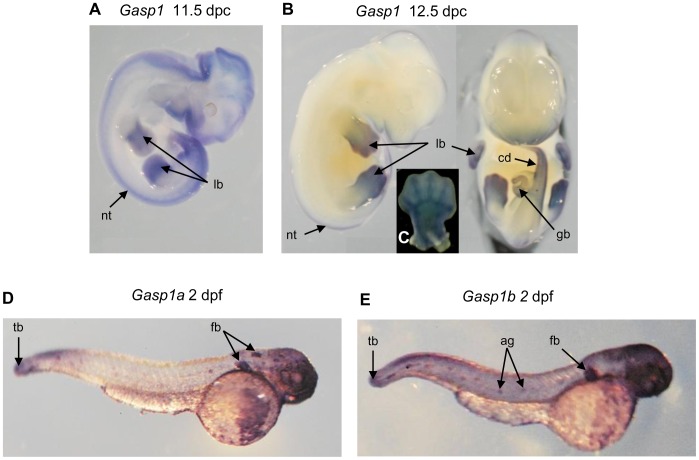
*Gasp1* expression patterns. A and B: *in situ* hybridization with a *Gasp1* probe on mouse embryos at 11.5 dpc and 12.5 dpc reveals expression in neural tube (nt), limb bud (lb), genital bud (gb) and caudal part of the embryo (cd). Enlargement of the limb bud (C) shows an expression in the precartilaginous condensations that give rise to fingers. D and E: *in situ* hybridization with *Gasp1* probes on zebrafish embryos at 2 dpf showing expression patterns in tail bud (tb), fin buds (fb) and angioblasts (ag).

The expression domains of *Gasp1a* and *Gasp1b* were also studied in zebrafish embryos at 2 dpf by *in situ* hybridization (Fig. 5). A similar expression of these two genes was observed in pectoral fin buds, tail bud, angioblasts (more obvious for *Gasp1a*) and the hindbrain anterior region, rostral to the optic vesicle, probably corresponding to extraocular musculature.

## Discussion

**Figure 6:**
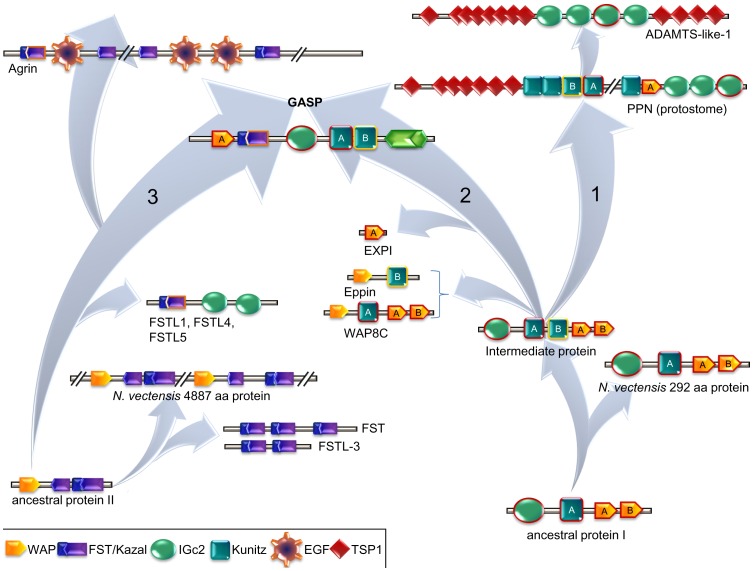
Global view of GASP/Papilin evolution. This representation derived from the phylogenies conducted on the separate modules (Fig. S1, S2, S3, and S4). From a protein close to the actual *N. vectensis* 292 aa protein (bottom right), an intermediate protein probably arose through a duplication of the kunitz domain. This form would represent a common ancestor to papilin (PPN, arrow 1) and GASP (arrow 2). The schematized papilin modular organization corresponds to that found in *Drosophila melanogaster* isoforms. The phylogeny of the IGc2 modules allowed us to situate ADAMTS protein close to papilin. The GASP kazal domain came from a donor polypeptide which could be agrin, FSTL1, 4, or 5 proteins. The FST and FSTL3 kazal domains are unlikely related to GASP module but very close to the units present in *N. vectensis* 4887 aa protein. Eppin, EXPI, and WAP8C are examples of proteins that probably evolved from the intermediate form. Domains related to *N. vectensis 292* aa protein are outlined in red, the new kunitz domain B (intermediate step) is outlined in yellow. The closest kazal domain between agrin, FSTL1, 4, 5 proteins and GASP is outlined in orange.

The origin of the modular structure of proteins is an interesting issue. According to the exon shuffling model [18], [19], it is necessary that every domain is encoded by a special exon [20] as it was seen in the sea urchin-acorn worm clade [15]. Finding one or more common modules is also important to establish relationships between proteins but the major prerequisite is to prove a phylogenetic relationship between sister proteins for each of these modules. In the sea anemone *Nematostella vectensis*, a primitive metazoan, we found two collinear genes that encoded most domains included in vertebrate GASP. This suggests that *Gasp* genes resulted from a recombination between the equivalent of two encoding sequences, as demonstrated for other genes [21]. To get insight into the events separating sequences included in primitive metazoans and deuterostome GASP, we studied the phylogeny of each module separately (WAP, kazal, IGc2, and both kunitz). The congruence of the different evolutionary trees produced suggests a common step between GASP and papilin (Fig. 6). Both proteins share close WAP, IGc2, and both categories of kunitz. As papilin sequence can be retrieved in bilateria, the divergence between GASP and papilin dates before the last common ancestor to this clade. Given the composition of the 292 aa protein present in *Nematostella* (IGc2, kunitz, and 2 WAPs), we hypothesize that the major event separating this protein from the papilin/GASP ancestral sequence is the duplication of kunitz. The addition of the kazal domain in GASP could have resulted from three candidates donors: tomoregulin 1 and 2, agrin, and FSTL (Fig. S2). As tomoregulin seems to be limited to vertebrates, FSTL1, 4, 5 to deuterostomes, and agrin to bilateria [22], we propose that kazal could have come from agrin or FSTL1, 4 or 5. Some proteins, such as EXPI and WAP8C, sharing one or two modules with GASP and papilin would have diverged later. As for FST and FSTL3 (3 and 2 kazal domains respectively), their sequences are close to the 4887 aa protein of *N. vectensis* suggesting that FST and FSTL3 are directly linked to the *Nematostella* sequence and have no common history with GASP, unlike FSTL1, FSTL4, and FSTL5.

The exon shuffling process continued by the addition of a sequence encoding the signal peptide in the 5′end. This view is supported by an intron still present in sea urchin. Finally, the total structure was completed by addition of a sequence encoding the NTR domain at the 3′ end. The ancestral complete sequence was probably acquired by early deuterostomes but we still must examine the genomes of organisms recently assigned to this lineage such as Acoelomorph flatworms in detail [15]. Later in chordates, we observed a progressive elimination of introns as there was only one remaining in mouse sequences. The significance of this loss is unclear as this phenomenon is usually associated with a high level of transcription [23] which does not seem to be the case, at least in adult *Mus musculus*. The long branch corresponding to *Ciona* could be linked to the loss of the IGc2 module and maybe NTR domains, suggesting a strong modification of its function. The divergence of *Gasp1* and *Gasp2* is attributable to the first round of whole genome duplication [24], [25] as (i) blocks of paralogues identified in humans correspond to a unique chromosome or contig in *Ciona* or *Branchiostoma*, and (ii) the position of lamprey sequences is definitely in the GASP2 group and not at the base of GASP1 and GASP2 groups.

The common origin of GASP and papilin could highlight their fundamental functions. As demonstrated by Kramerova *et al.* [26], papilin mostly studied in *D. melanogaster* is secreted and localized in the basal membrane. In adults, this protein is expressed in Malpighian tubules, salivary glands, trachea, and muscles with their innervations. Papilin overexpressing flies show abnormalities in muscle cells, tracheae, and Malpighi tubules. RNAi experiments conducted on drosophila embryos led to frequent decreases in survival rates, linked to muscle defects but without consequences on tracheae, and Malpighi tubules. Through its extracellular matrix localization, it has been suggested that papilin influences cell rearrangements in muscle tissue [26], [27]. This probable function gives new perspectives for analysing GASP activity in muscle development.

*In vitro* experiments showed that GDF8 and GDF11 had a greater affinity toward GASP1 than GASP2 although surface plasmon resonance measurements indicated a slightly stronger relationship between GDF8 and GASP1 on one hand, and between GDF11 and GASP2 on the other hand [4]. Sequence conservation in peptidic modules of mammalian GASP1 and GASP2 is probably involved in protein-protein interaction. As the entire GASP1sequence is more conserved than GASP2, we could hypothesize that there is a strong partnership between GASP1 and both GDF8 and GDF11 proteins. This observation was verified for every protein module except for the C-terminal portion of IGc2. GASP proteins contained several domains (WAP, kazal, kunitz, and maybe NTR) described in protease inhibitors conferring them with a potential antiproteasic activity. It does not seem that the WAP domain is important in this respect given its high substitution rate. In contrast, the follistatin/kazal unit in GASP1 shows the same low level of substitution as in the follistatin protein, indicating that this GASP1 module could be functional compared to the one present in GASP2. A similar situation was found in the first kunitz in GASP1 and GASP2. However, for the netrin domain, both substitution rate curves overlapped, suggesting the same activity. The IGc2 domain could have a structural function in protein conformation supported by its high sequence conservation. Interactions between GDF8/GDF11 and GASP1/GASP2 indicate that the ancestors of both groups of proteins shared this relationship before the emergence of vertebrates.

Although weak signals were detected by *in situ* hybridization, our data suggest common embryonic expression domains between *Gasp1*, *Gdf8* and *Gdf11*. McPherron *et al.* [5] have described *Gdf8* expression from 9.5 dpc on in developing somites. *Gdf11* was expressed from 9.5 to 13.5 dpc in more caudal parts [6], [28]. Our experiments could not precisely indicate the tissue type associated with *Gasp1* expression in mouse limb buds. Co-localization assays using *Gdf8* and *Gdf11* probes may help to a better characterization as well as *Sox9* which could be useful to mark precartilagenous condensations [29].

We observed in mammals that the linker region between kazal and IgC2 domains or IgC2 and kunitz modules present more substitutions in GASP2 than in GASP1. As a result, these substitutions may have affected the flexibility and folding of mammal GASP2 protein.

**Figure 7:**
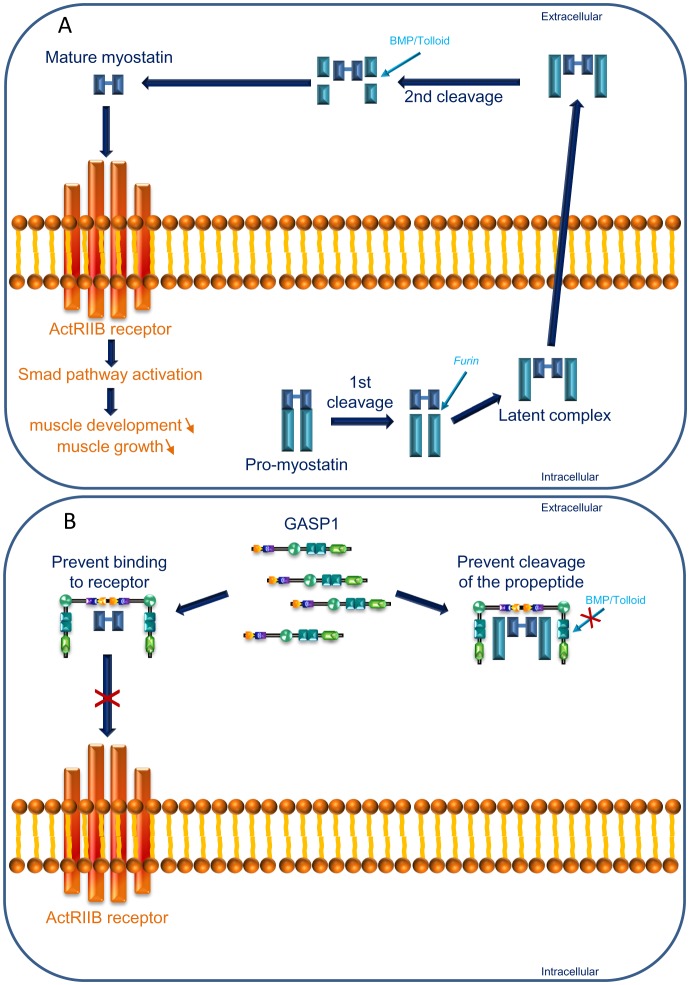
Model of interaction between GASP1 and Myostatin. (after Lee, 2004) A) Myostatin signalling through ActRIIB receptor is crucial for the regulation of muscle growth. Myostatin is synthesized as a precursor protein (pro-myostatin) that undergoes proteolytic processing (furin cleavage) to generate an N-terminal propeptide and a disulfide-linked C-terminal dimer, which is the biologically active molecule. Myostatin is in circulation as an inactive latent complex (propeptide and mature myostatin) which is activated by BMP/Tolloid family of metalloproteinases. Active myostatin, mostly binds to the ActRIIB receptor and engages the signalling cascade leading to the inhibition of myoblast differentiation and proliferation. B) Specific inhibitors can prevent binding of myostatin to ActRIIB. One of them is GASP1 which also binds to the propeptide. We hypothezise that GASP1 could interact with the inactive latent complex preventing propeptide cleavage by BMP/Tolloid through antiprotease activity of the second kunitz domain.

Finally, based on the review of Lee [30], we present a synthetic view (Fig. 7) illustrating possible interactions between GASP1 and GDF8 (myostatin). When GDF8 is purified from plasma [2], it is present as a homodimer. Furthermore, it has been shown that GASP1 binds to GDF8 in a 1∶1 ratio by *in vitro* experiments [4]. We propose that two molecules of GASP1 could bind to GDF8 alone or to the inactive latent complex, protecting it from proteolysis through the kunitz domains (mainly the second one) and from binding to the ActRIIB receptor, leading to an inhibition of myoblast proliferation and differentiation.

Our model needs to be challenged by *in vitro* experiments on direct interaction between the two partners. Symmetric analysis dealing with GASP2 could also reinforce our knowledge of the complex situation between GASP1/GASP2 and GDF8/GDF11.

## Materials and Methods

### Ethics Statement

All experimental animal procedures were carried out in accordance with the recommendations in the guidelines of the European Communities Council (Directive 86/609EC). Experiments were approved by the committee on the ethics of animal experiments of the author’s institution, “Comité Régional d’Ethique de l’Expérimentation Animale” of the Limousin region (CREEAL N°33).

### Sequences

Only metazoan sequences were considered for this study. Orthologous GASP proteins were identified from all genomic and EST sequences available in the general databases such as NCBI and ENSEMBL for most organisms. We also explored specialized databases, as JGI for the amphioxus *Branchiostoma floridae* and *Strongylocentrotus purpuratus*, the Genome Sequencing Center at the Washington University School of Medicine (St Louis MO) for the sea lamprey *Petromyzon marinu*s, and the Human Genome Sequencing Center at Baylor College of Medicine for the Acorn worm *Saccoglossus kowaleskii* (http://blast.hgsc.bcm.tmc.edu/blast.hgsc?organism  = 20), using BLASTP, TBLASTN with default parameters (an e-value cut off at 0.01 was used in all BLAST searches). New complete open reading frames were annotated and submitted to EMBL/GenBank as putative *Gasp* sequences. The search of sequences sharing different module combinations with GASP was performed using SMART [31], [32], (http://smart.embl-heidelberg.de/). We included in the analysis all mouse proteins having one module in common with GASP and extended to all organisms for proteins sharing at least two modules with GASP. The identification of different modules (limits and sequences) was done using SMART with PFAM and signal peptide options.

### Phylogenetic Analysis

The alignment of amino acid sequences (see Tables S1, S2, S3 for access numbers) was conducted using ClustalW 2.0 software [33]. The informative positions within protein alignments were selected by Gblocks 0.91b with the options of less stringent selection [34]. Maximum likelihood (ML) and Minimum Evolution (ME) analyses were done with MEGA 5.05 [35]. The model of protein evolution that best fits a given set of aligned sequences was chosen according to the calculations provided by ProtTest 3.0 [12]. Bootstrap values for the nodes were determined by analyzing 500 or 2000 replicates for ML and ME respectively.

### Synteny Analysis and Paralogon Detection

Synteny and orthology between human and invertebrates *Gasp* genes were assessed by using synteny database program [17] with a window of 50 to 100 genes. The paralogy between human chromosomes bearing *Gasp1* and *Gasp2* was also confirmed by another algorithm implemented in paralogon website [16].

### Substitution Rates

The substitution number per site from *Ciona* to tetrapods on the one hand, and to fish on the other hand (sequences in Tables S1, S2 and S3) and during mammal evolution (23 species for GASP1, 25 for GASP2 and 22 for follistatin; see Tables S1, S2, and S4 respectively) was determined using the methodology described in Petit *et al.* [36] and Martin *et al*. [37]. Briefly, the parenthetic topology obtained with MEGA 5.0 and PHYML was taken as user tree for running the parsimony program Protpars included in the PHYLIP Package [38]. The obtained substitution numbers per site were plotted in parallel with the structural domains of both GASP proteins.

### *In Situ* Hybridization

In zebrafish, probe synthesis and *in situ* hybridization were carried out as previously described by Hammerschmidt *et al.* [39]. Sense and antisense probes were synthesized with DIG RNA labelling kit (Roche) from brain cDNA amplified by the following primers: ZfGasp1aFwd: AATACCAGCCCTCTCCCAGT and ZfGasp1aRev: TGAAGGATGAGAAGATGGGG for the 930 nucleotide Gasp1a probes (GenBank accession number: JQ266018); ZfGasp1bFwd: GCTTTACCAACAACCGCATT and ZfGasp1bRev: CTGGTCATGGTGGAGGAGATA for the 861 nucleotide Gasp1b probes (GenBank accession number: JQ266019). Zebrafish brain cDNA and zebrafish embryos were generously provided by Dr. L. Bally Cuif (CNRS Zebrafish Neurogenetics laboratory, Gif sur Yvette, France).

Mouse embryos were dissected in PBS and fixed overnight in 4% paraformaldehyde at 4°C. The antisense 595 nucleotide *Gasp1* probe was synthesized with DIG RNA labelling kit (Roche) from muscle cDNA amplified by the following primers: Gasp595-fwd: GCTCACTTGGGAGAAACAGC and Gasp595-rev: GAAGGGACACGACTCCTCAC.

RNA *in situ* analysis was performed essentially as described [40] except that NBT (4.5 µl/ml)/BCIP (3.5 µl/ml) in NTMT were used as the AP substrate.

## Supporting Information

Figure S1 **Phylogenetic analysis of the WAP domain.** The tree was constructed using maximum likelihood method with JTT+G options. Blue names indicate mouse proteins, black names correspond to other organism proteins. The subtree including the 292 aa and 4887 aa proteins present in *Nematostella vectensis* is in green, the subtree containing GASP in red. When several WAP domains are present in a single protein, each module is named according to its position.
(TIF)

Figure S2 **Phylogenetic analysis of the kazal domain.** The tree was constructed using maximum likelihood method with WAG+G options. Blue names indicate mouse proteins, black names correspond to other organism proteins. The subtree including the 4887 aa protein present in *Nematostella vectensis* is in green, the subtree containing GASP in red. When several kazal domains are present in a single protein, each is named according to its position.
(TIF)

Figure S3 **Phylogenetic analysis of the IGc2 domain.** The tree was constructed using maximum likelihood method with WAG+G options. Blue names indicate mouse proteins, black names correspond to other organism proteins. The subtree including the 292 aa protein present in *Nematostella vectensis* is in green, the subtree containing GASP in red. When several IGc2 domains are present in a single protein, each is named according to its position. The first IGc2 domain of *drosophila melanogaster* papilin (PPN-1) was removed because of its high divergency.
(TIF)

Figure S4 **Phylogenetic analysis of the kunitz domains.** The tree was constructed using maximum likelihood method with WAG+G options. Blue names indicate mouse proteins, black names correspond to other organism proteins. The subtree including the 292 aa protein present in *Nematostella vectensis* is in green, the subtree containing GASP in red. When several kunitz domains are present in a single protein, each is named according to its position.
(TIF)

Table S1 **Access number of GASP1 proteins.** Access numbers beginning with “EN” are from ENSEMBL, the others from NCBI.
(DOC)

Table S2 **Access number of GASP2 proteins.** Access numbers beginning with “EN” are from ENSEMBL, the others from NCBI.
(DOC)

Table S3 **Access number or localisation of GASP proteins.** SPU_004017 is an ID from SpBase (www.spbase.org).
(DOC)

Table S4 **Access number of the follistatin protein.** Access numbers beginning with “EN” are from ENSEMBL, the others from NCBI.
(DOC)
