# Supplementary figures and images for: GASP/WFIKKN Proteins: Evolutionary Aspects of Their Functions
Source: PLoS One. 2012 Aug 24;7(8):e43710. doi: 10.1371/journal.pone.0043710 (PMC3427181; doi:10.1371/journal.pone.0043710)

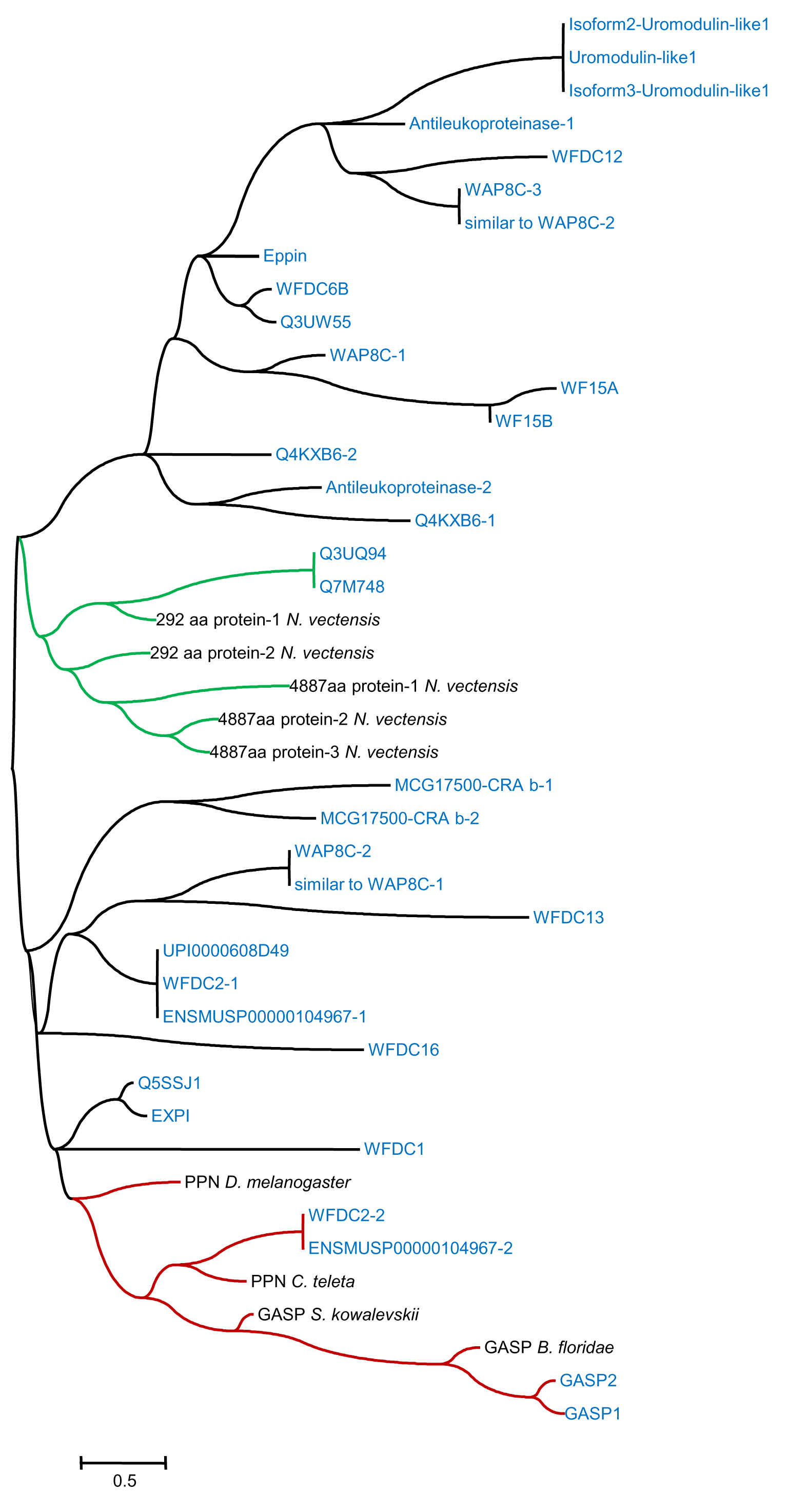

Supplement: Figure S1 — Phylogenetic analysis of the WAP domain. The tree was constructed using maximum likelihood method with JTT+G options. Blue names indicate mouse proteins, black names correspond to other organism proteins. The subtree including the 292 aa and 4887 aa proteins present in Nematostella vectensis is in green, the subtree containing GASP in red. When several WAP domains are present in a single protein, each module is named according to its position. (TIF) [file pone.0043710.s001.tif]

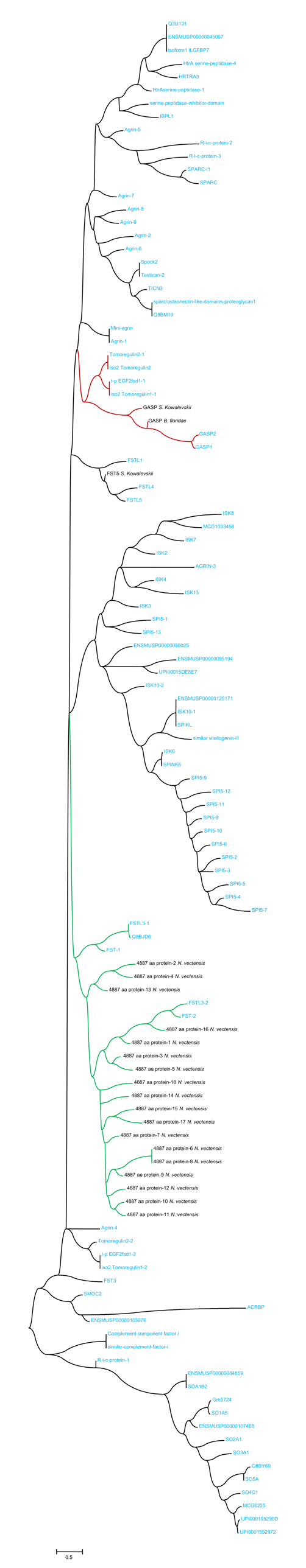

Supplement: Figure S2 — Phylogenetic analysis of the kazal domain. The tree was constructed using maximum likelihood method with WAG+G options. Blue names indicate mouse proteins, black names correspond to other organism proteins. The subtree including the 4887 aa protein present in Nematostella vectensis is in green, the subtree containing GASP in red. When several kazal domains are present in a single protein, each is named according to its position. (TIF) [file pone.0043710.s002.tif]

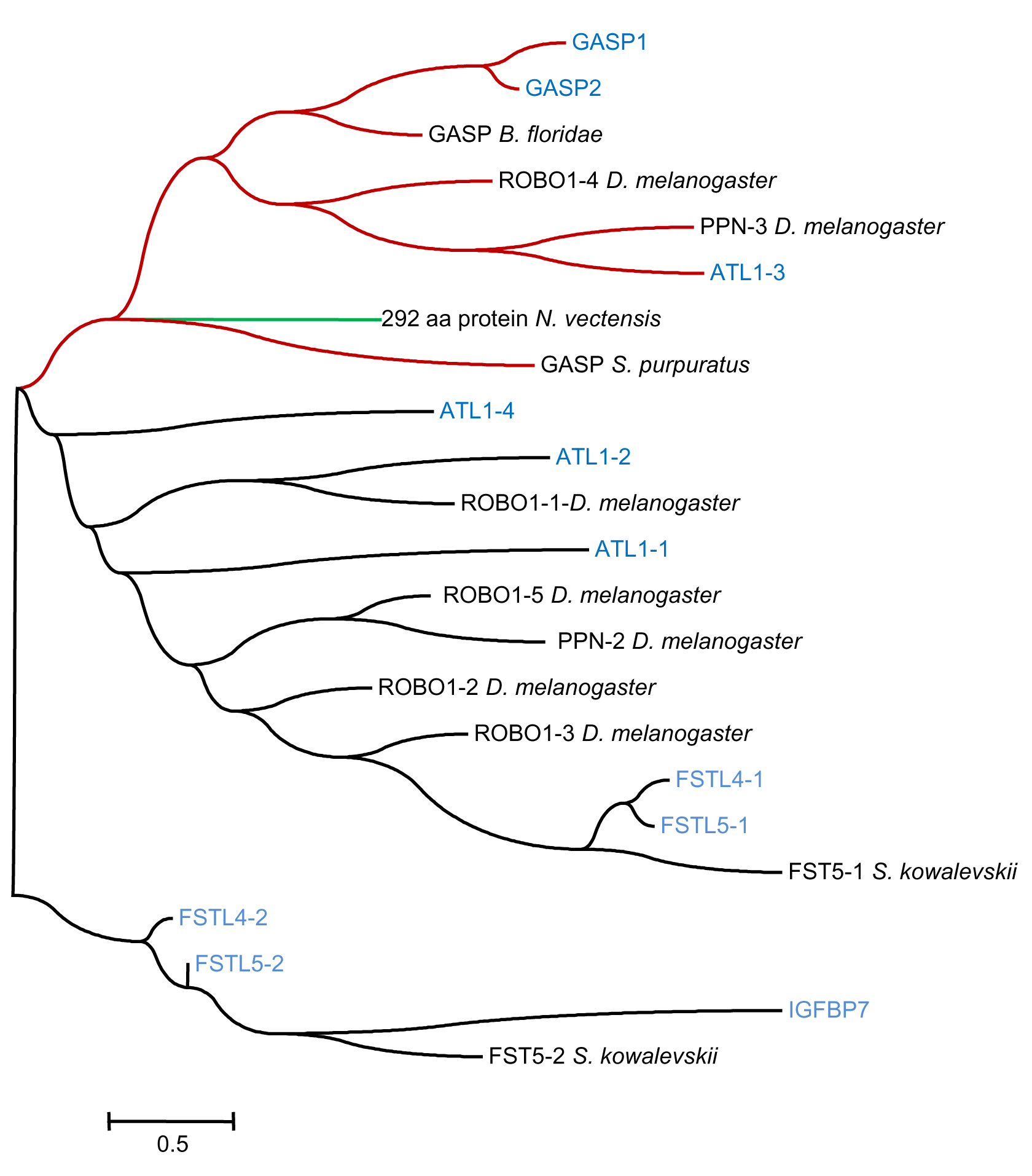

Supplement: Figure S3 — Phylogenetic analysis of the IGc2 domain. The tree was constructed using maximum likelihood method with WAG+G options. Blue names indicate mouse proteins, black names correspond to other organism proteins. The subtree including the 292 aa protein present in Nematostella vectensis is in green, the subtree containing GASP in red. When several IGc2 domains are present in a single protein, each is named according to its position. The first IGc2 domain of drosophila melanogaster papilin (PPN-1) was removed because of its high divergency. (TIF) [file pone.0043710.s003.tif]

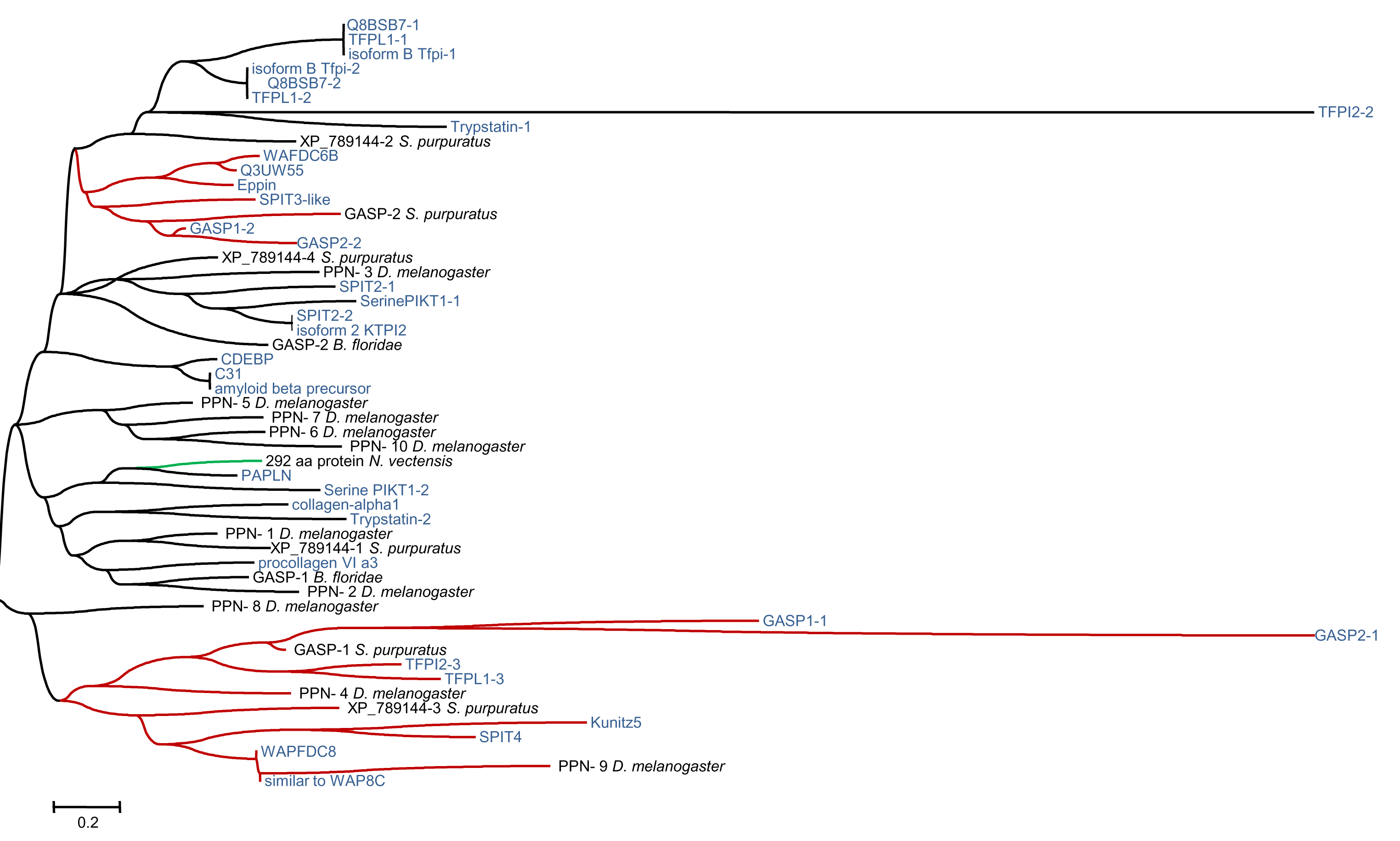

Supplement: Figure S4 — Phylogenetic analysis of the kunitz domains. The tree was constructed using maximum likelihood method with WAG+G options. Blue names indicate mouse proteins, black names correspond to other organism proteins. The subtree including the 292 aa protein present in Nematostella vectensis is in green, the subtree containing GASP in red. When several kunitz domains are present in a single protein, each is named according to its position. (TIF) [file pone.0043710.s004.tif]
