# Supplementary material for: GASP/WFIKKN Proteins: Evolutionary Aspects of Their Functions
Source: PLoS One. 2012 Aug 24;7(8):e43710. doi: 10.1371/journal.pone.0043710 (PMC3427181; doi:10.1371/journal.pone.0043710)
Supplement: Table S1 — Access number of GASP1 proteins. Access numbers beginning with “EN” are from ENSEMBL, the others from NCBI. (DOC) [file pone.0043710.s005.doc]

**Table S1. Access number of GASP1 proteins.**

| **Animal** | **GASP1 protein access number** |
| --- | --- |
| Anole lizard (*Anolis carlinensis*) | ENSACAP00000001579 |
| Armadillo (*Dasypus novemcinctus*) | ENSDNOP00000015656 |
| Bushbaby (*Otolemur garnettii*) | ENSOGAP00000007961 |
| Cat (*Felis catus*) | ENSFCAP00000011455 |
| Chiken (*Gallus gallus* | ENSGALP00000011904 |
| Cow (*Bos taurus*) | DAA18604.1 |
| Dog (*Canis familiaris*) | XP_548206.2 |
| Dolphin (*Tursiops truncatus*) | ENSTTRP00000002564 |
| Elephant (*Loxodonta africana*) | ENSLAFP00000029291 |
| Fugu (*Takifugu rubripes*) a | ENSTRUP00000021730 |
| Fugu (*Takifugu rubripes*) b | ENSTRUP00000008030 |
| Gorilla (*Gorilla gorilla*) | ENSGGOP00000019008 |
| Guinea pig (*Cavia porcellus*) | ENSCPOP00000019147 |
| [Hedgehog (*Erinaceus europaeus*)](http://en.wikipedia.org/wiki/European_Hedgehog) | ENSEEUP00000003809 |
| Horse (*Equus caballus*) | XP_001499830.1 |
| Human (*Homo sapiens*) | NP_783165.1 |
| Kangaroo rat (*Dipodomys ordii*) | ENSDORP00000013011 |
| Lesser hedgehog tenrec (*Echinops telfairi*) | ENSETEP00000003334 |
| Macaque (*Macaca mulatta*) | XP_001100200.1 |
| Marmoset (*Callithrix jacchus*) | XP_002748468.1 |
| Mouse (*Mus musculus*) | NP_861540.2 |
| Mouse lemur (*Microcebus murinus*) | ENSMICP00000001752 |
| Opossum (*Monodelphis domestica*) | XP_003340244.1 |
| Orangutan (*Pongo abelii*) | ENSPPYP00000009295 |
| Panda (*Ailuropoda melanoleuca*) | XP_002926025.1 |
| Pig (*Sus scrofa*) | ENSSSCP00000018606 |
| Pika (*Ochotona princeps*) | ENSOPRP00000012362 |
| Rabbit (*Oryctolagus cuniculus*) | ENSOCUP00000000344 |
| Tree Shrew (*Tupaia belangeri*) | ENSTBEP00000002924 |
| *Xenopus tropicalis* | ENSXETP00000054046 |
| Zebrafish (*Danio rerio*) a | ENSDARP00000025648 |
| Zebrafish (*Danio rerio*) b | ENSDARP00000076606 |

Access number beginning with “EN” are from ENSEMBL, the others from NCBI.
