# Supplementary material for: GASP/WFIKKN Proteins: Evolutionary Aspects of Their Functions
Source: PLoS One. 2012 Aug 24;7(8):e43710. doi: 10.1371/journal.pone.0043710 (PMC3427181; doi:10.1371/journal.pone.0043710)
Supplement: Table S2 — Access number of GASP2 proteins. Access numbers beginning with “EN” are from ENSEMBL, the others from NCBI. (DOC) [file pone.0043710.s006.doc]

**Table S2.** **Access number of GASP2 proteins.**

| **animal** | **GASP2 protein access number** |
| --- | --- |
| Anole lizard (*Anolis carlinensis*) | ENSACAP00000012461 |
| Bushbaby (*Otolemur garnettii*) | ENSOGAP00000004833 |
| Chiken (*Gallus gallus*) | XP_426858.2 |
| Cow (*Bos taurus*) | NP_001192725.1 |
| Dog (*Canis familiaris*) | XP_547215.3 |
| Dolphin (*Tursiops truncatus*) | ENSTTRP00000008750 |
| Elephant (*Loxodonta africana*) | ENSLAFP00000023783 |
| Fugu (*Takifugu rubripes*) 1 | ENSTRUP00000036143 |
| Gorilla (*Gorilla gorilla*) | ENSGGOP00000007720 |
| Guinea pig (*cavia porcellus*) | XP_003478465.1 |
| Horse (*Equus caballus*) | XP_001495753.1 |
| Human (*Homo sapiens*) | NP_444514.1 |
| Hyrax (*Procavia capensis*) | ENSPCAP00000014761 |
| Kangaroo rat (Dipodomys ordii) | ENSDORP00000001896 |
| Lesser hedgehog tenrec (*Echinops telfairi*) | ENSETEP00000004628 |
| Macaque (*Macaca mulatta*) | ENSMMUP00000028924 |
| Marmoset (*Callithrix jacchus*) | ENSCJAP00000021972 |
| Megabat (*Pteropus vampyrus*) | ENSPVAP00000000531 |
| Microbat (*Myotis lucifugus*) | ENSMLUP00000009356 |
| Mouse (*Mus musculus*) | NP_001093924.1 |
| Opossum (*Monodelphis domestica*) | XP_003341725.1 |
| Orangutan (*Pongo abelii*) | ENSPPYP00000007830 |
| Panda (A*iluropoda melanoleuca*) | XP_002920208.1 |
| Rat (*Rattus norvegicus*) | XP_001081284.2 |
| Sea lamprey (*Petromizon marinus*) a | ENSPMAP00000006686 |
| Sea lamprey (*Petromizon marinus*) b | ENSPMAP00000009096 |
| Shrew (*Sorex araneus*) | ENSSARP00000009732 |
| Wallaby (*Macropus eugenii*) | ENSMEUP00000008614 |
| *Xenopus tropicalis* | ENSXETP00000043703 |
| Zebrafish (*Danio rerio*) | ENSDARP00000098282 |
|  |  |

Access number beginning with “EN” are from ENSEMBL, the others from NCBI.
