# Supplementary material for: GASP/WFIKKN Proteins: Evolutionary Aspects of Their Functions
Source: PLoS One. 2012 Aug 24;7(8):e43710. doi: 10.1371/journal.pone.0043710 (PMC3427181; doi:10.1371/journal.pone.0043710)
Supplement: Table S3 — Access number or localisation of GASP proteins. SPU_004017 is an ID from SpBase (www.spbase.org). (DOC) [file pone.0043710.s007.doc]

**Table S3.** **Access number or localisation of GASP proteins.**

| **Animal** | **GASP protein access number or localisation** |
| --- | --- |
| *Bramchiostoma floridae* | Bfl-V2-48 |
| *Ciona intestinalis* | Chr 10q (cDNA: AK112345) |
| *Ciona Savignyi* | cont_41805 |
| *Nematostella vectensis* | Scaffold 86 |
| *Saccoglossus kowalevskii* | XP_002732704.1 |
| *Strongylo purpuratus* | SPU_004017 |

SPU_004017 is an ID from SpBase ([www.spbase.org](http://www.spbase.org/)).
