# Supplementary material for: GASP/WFIKKN Proteins: Evolutionary Aspects of Their Functions
Source: PLoS One. 2012 Aug 24;7(8):e43710. doi: 10.1371/journal.pone.0043710 (PMC3427181; doi:10.1371/journal.pone.0043710)
Supplement: Table S4 — Access number of the follistatin protein. Access numbers beginning with “EN” are from ENSEMBL, the others from NCBI. (DOC) [file pone.0043710.s008.doc]

**Table S4. Access number of the follistatin protein.**

| **Animal** | **Follistatin protein access number** |
| --- | --- |
| Alpaca (*Vicugna pacos*) | ENSVPAP00000001038 |
| Cat (*Felis catus*) | ENSFCAP00000002232 |
| Chinese hamster (*Cricetulus griseus*) | EGV92181.1 |
| Cow (*Bos taurus*) | AAA30522.1 |
| Dolphin (*Tursiops truncatus*) | ENSTTRP00000011717 |
| Elephant (*Loxodonta africana*) | ENSLAFP00000008993 |
| [Goat (*Capra hircus*)](http://www.ncbi.nlm.nih.gov/protein/ADN03390.1) | ADN03390.1 |
| Gorilla (*Gorilla gorilla*) | ENSGGOP00000002927 |
| [Hedgehog (*Erinaceus europaeus)*](http://en.wikipedia.org/wiki/European_Hedgehog) | ENSEEUP00000012815 |
| horse (*Equus caballus*) | ENSECAP00000015558 |
| Human (*Homo sapiens*) | AAH04107.1 |
| Macaque (*Macaca mulatta*) | EHH26486.1 |
| Megabat (*Pteropus vampyrus*) | ENSPVAP00000013195 |
| Mouse (*Mus musculus*) | CAA82648.1 |
| Opossum (*Monodelphis domestica*) | ENSMODP00000024281 |
| Pig (*Sus scrofa*) | CAH05035.1 |
| Pika *(Ochotona princeps*) | ENSOPRP00000006046 |
| Rabbit *(Oryctolagus cuniculus)* | ENSOCUP00000004047 |
| Ray (*Rattus norvegicus*) | AAB60704.1 |
| Squirel (*Spermophilus tridecemlineatus*) | ENSSTOP00000011893 |
| Wallaby (*Macropus eugenii*) | ENSMEUP00000004754 |
| Water buffalo (*Bubalus bubalis*) | ABQ96267.1 |

Access number beginning with “EN” are from ENSEMBL, the others from NCBI
